# Percutaneous closure of veno-venous collaterals in adult patients with univentricular physiology after Fontan palliation: Single centre experience and systematic review

**DOI:** 10.1016/j.ijcchd.2023.100479

**Published:** 2023-10-11

**Authors:** Marieke Nederend, Anastasia D. Egorova, Frank van der Kley, Philippine Kiès, Arno A.W. Roest, Martin J. Schalij, Monique R.M. Jongbloed

**Affiliations:** a CAHAL, Center for Congenital Heart Disease Amsterdam Leiden, Leiden University Medical Center, Leiden, the Netherlands; b Department of Cardiology, Leiden University Medical Center, Leiden, the Netherlands; c Department of Paediatrics, Division of Paediatric Cardiology, Leiden University Medical Center, Leiden, the Netherlands; d Department of Anatomy & Embryology, Leiden University Medical Center, Leiden, the Netherlands

**Keywords:** Adult congenital heart disease, Fontan circulation, Veno-venous collaterals, Univentricular heart, Long term complications, Transcatheter interventions

## Abstract

**Background:**

The Fontan operation resulted in improved survival of patients with congenital heart defects not equipped to sustain biventricular circulation. Long-term complications are common, such as veno-venous collaterals (VVC). The aim of this study was to evaluate patient characteristics, percutaneous treatment strategy and (short-term) outcomes in adult Fontan patients with VVC, and review literature to date.

**Methods:**

In this single-centre retrospective observational cohort study, patients who underwent percutaneous VVC closure between 2017 and 2023 were identified.

**Results:**

Thirteen patients underwent percutaneous VVC closure (77 % female, age at intervention 24 ± 4 years, 77 % systemic left ventricle, 77 % extracardiac tunnel, median conduit size 16 [16–20]mm). Indications for closure were symptoms and/or significant exercise-related hypoxia. Mean Fontan pressure was 10±4 mmHg. The VVC originated from tributaries of the vena cava superior (VCS) and connected to pulmonary veins (8 VVC, 32 %), VCS to systemic atrium (3 VVC, 12 %), VCS to coronary sinus (3 VVC, 12 %) and tributaries of vena cava inferior to pulmonary veins (11 VVC, 44 %). Twenty-three VVC were occluded using coils and/or plugs. No periprocedural complications occurred. At first follow-up at least 6 months after closure (n = 11), 9 patients (82 %) reported symptom reduction. Saturation at rest and peak exercise increased significantly (96 ± 3 to 98 ± 1 %, p = 0.040; 89 ± 3 to 93 ± 5 %, p = 0.024, respectively). Exercise capacity remained unchanged.

**Conclusions:**

VVC typically connect the tributaries of the vena cava inferior and/or superior with the pulmonary veins. Low Fontan pressures do not exclude the presence of VVC. Percutaneous closure of VVC is technically feasible, safe, and associated with symptom reduction and a significant rise in resting and exercise oxygen saturation.

## 1 Introduction

At the most severe end of the congenital heart disease spectrum are patients with an univentricular physiology, comprising a heterogenous group of congenital heart malformations that have the common characteristic that the cardiac condition is not equipped for sustaining a biventricular circulation [[1], [2], [3]]. With a peri-operative mortality of <5 % and 10-year survival approaching 90 %, the adult population of patients with a Fontan circulation is growing [[4], [5], [6], [7]]. This increase in survival is, however, inevitably accompanied by long term complications affecting multiple organ systems, resulting in gradual decline in cardiovascular performance at adult age [4,5,8,9].

Veno-venous collaterals (VVC) are commonly encountered sequelae, this entails collaterals forming between the systemic venous return and the pulmonary venous return tributaries [4]. These result in a reduced arterial oxygen saturation. It is speculated that VVC can have both potentially beneficial (unloading of Fontan circulation in the form of a natural fenestration and increase of cardiac output due to right to left shunting) and adverse physiological effects (cyanosis resulting in hypoxia induced pulmonary vasoconstriction and rise in Fontan filling pressures). The prevalence and the optimal management strategy of VVC in patients with (functional) univentricular heart disease after Fontan palliation (Fontan patients) remains controversial with often contradicting results reported in the literature [[10], [11], [12]]. The guidelines specify that resting and/or exertional hypoxemia might be a result of VVC and therefore further evaluation is recommended. An individual case-by-case approach to treatment strategy of VVC, taking into consideration the balance between cyanosis and the possible negative hemodynamic effects is advised [4,5]. In light of these recommendations, and given the large heterogeneity in anatomical substrate, as well as the still relatively limited follow-up in adult patients with VVC after Fontan palliation, in the current study we aim to (1) evaluate patient characteristics, the percutaneous treatment strategy and (short-term) outcomes in adult Fontan patients with VVC (2) , to assess the reported prevalence of VVC and (3) to put these findings in perspective of current knowledge by evaluating treatment strategies and outcomes as described in literature to date.

## 2 Methods

### 2.1 Design and inclusion/exclusion criteria

This single-centre, retrospective descriptive cohort study was performed at the department of Cardiology of the Center for Congenital Heart Disease Amsterdam Leiden (CAHAL), location Leiden University Medical Center (LUMC). All consecutive adult patients with a Fontan circulation under follow-up at our centre were evaluated. Patients with an open fenestration were excluded. Patients who underwent percutaneous closure of a VVC were selected and included for further analysis. Indications for closure were symptoms and/or significant exercise-related hypoxia.

### 2.2 Data collection and follow-up

Clinical and demographic data were collected from electronical patient records. The closing date for follow-up was June 2023. All the reported follow-up visits and investigations were performed as part of routine clinical care. For the complete patient cohort, patient characteristics, including medical history, were collected. Age, height and weight were assessed at the most recent follow-up moment before closing date of follow-up for the study. For the patients who underwent percutaneous closure of a VVC data was collected at baseline (most recent outpatient clinic visit prior to percutaneous closure procedure), (peri-)procedural, and at the first outpatient clinic visit at least 6 months after the procedure. Data collected included: medical history, complaints, pharmacological therapy, physical examination, transthoracic echocardiography, magnetic resonance imaging (if available within 3 years of procedure), laboratory investigations (including haemoglobin, renal function, hepatic panel, and N-terminal-pro hormone brain natriuretic peptide (NT-proBNP)) and bicycle ergometry with VO_2_max including peripheral saturation at ambient air at rest and at peak exercise. Patient-reported symptoms were evaluated through subjective interviews with their treating cardiologist. Fontan associated liver disease was defined as present when a diagnosis of liver fibrosis or liver cirrhosis by the gastroenterologist was made based on abdominal imaging and/or FibroScan. Additionally, non-invasive liver fibrosis scores were calculated as follows: ASAT to platelet ratio index (APRI) = ([ASAT (U/L)/40 (U/L)]/platelet count [109/L])*100, and Fibrosis-4 Index (FIB-4) = (age [years]*ASAT [U/L])/(platelet count [109/L]*√ALAT [U/L]), where ASAT is aspartate transaminase and ALAT is alanine transaminase [13,14]. Echocardiograms were performed with commercially available ultrasound systems and were analysed offline in Echo PAC (GE Medical Systems, USA) by cardiologists with dedicated expertise in congenital imaging.

For the catheterization procedure, venous access sites, and anaesthetic conditions (defined as general anaesthesia, conscious sedation, or local anaesthesia) were reported. Furthermore, hemodynamic measurements were collected, including right-sided pressures and venous saturations. Fontan pathway obstruction was defined as a gradient above 2 mmHg between the vena cava inferior and Fontan conduit at rest [10]. Conventional selective and non-selective angiography was used to identify the VVC, and data on closure techniques, residual shunts and complications were collected. A collateral was designated as large if the diameter at selective angiography was ≥ 4 mm.

### 2.3 Outcomes

The primary outcome parameter was to evaluate the patient characteristics and the percutaneous treatment strategy in adult Fontan patients. Secondary outcome parameters consisted of clinical outcomes, including the change in complaints, peripheral oxygen saturation at rest and at peak exercise, and laboratory findings at least 6 months after the closure of VVC.

### 2.4 Systematic review

**Fig. 1:**
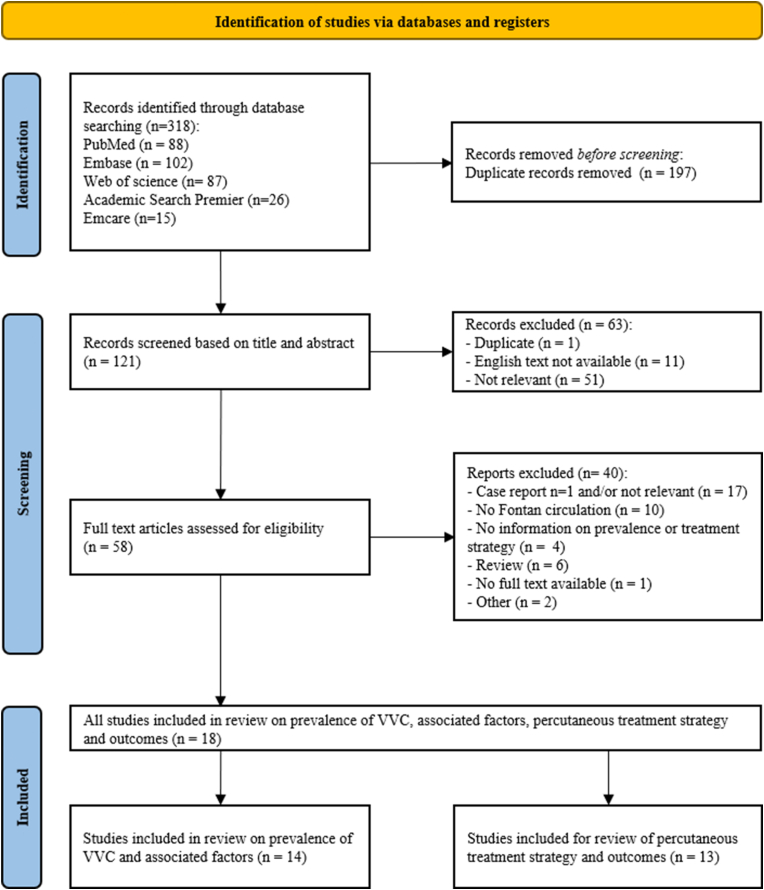
Prisma flow diagram illustrating the selection of studies on prevalence of VVC, associated factors, percutaneous closure strategy and outcomes VVC: veno-venous collaterals.

To correlate findings of our study with current literature, a comprehensive literature review was performed with the subjects 1. “Fontan circulation”, and 2. “veno-venous collaterals”, using the search engines PubMed, Embase, Web of Science, Cochrane Library and Emcare, see Supplement 1 for the complete queries. Two authors (M.N. and M.R.M.J.) independently screened and reviewed the articles on eligibility in a title and abstract phase and a full-text phase. Articles had to be available in full text and English language. Articles with inclusion of patients with a Fontan circulation, and investigation on the prevalence of VVC with or without treatment strategy and outcomes were included. Case reports and series with more than one patient reported were also included in the review for treatment and outcome. The article selection process is schematically shown in Fig. 1. Author M.N. conducted the data extraction process, collecting study details, baseline characteristics, and prevalence, clinical endpoints and associated factors. The outcome change in saturation was extracted if available and the mean difference and its 95 % confidence interval were calculated for each study.

### 2.5 Ethics statement

All tests and procedures performed involving human participants were in accordance with the ethical standards of the institutional and/or national research committee and with the 2013 Helsinki declaration or comparable ethical standards. Appropriate local scientific board approval was obtained and the need for written informed consent was waived by the institutional medical ethical board (protocol number: 2023-004). All patients provided consent for registration of their data and publication.

### 2.6 Statistical analysis

All statistical analyses were performed in IBM SPSS version 25. Normally distributed continuous data are displayed as mean ± standard deviation (SD) and non-normally distributed continuous data are displayed as median with the first and third quartile [Q1–Q3]. Proportions are displayed as numbers (percentages). Normality was graphically assessed and additionally tested with the use of the Shapiro-Wilk test. For the comparison between cohorts, an unpaired *t*-test was used for the continuous data and the chi-square test for the categorical data. For the comparison of continuous data between baseline and follow-up, a paired samples *t*-test was used. For categorical data, the McNemar test or Wilcoxon signed rank test was used, as appropriate. In case of substantial right skew in the outcomes, the natural log transformation was first applied. For the Forest plot, the mean difference and 95 % confidence interval in saturation was adjusted for the small study samples [[15], [16], [17]]. A p-value of <0.05 was considered statistically significant.

## 3 Results

### 3.1 Patient characteristics

A total of 49 adult Fontan patients without an open fenestration were identified. Patient characteristics are shown in Table 1. Of these patients, a total of 13 adult Fontan patients (13/49: 27 %) underwent percutaneous closure of VVC. There was a higher proportion of patients with a history of Fontan-associated liver disease in the group of Fontan patients who underwent percutaneous closure of VVC compared to the group of Fontan patients without closure procedure at adult age (54 % vs 19 %, p = 0.019). There was no differential for the demographic and anatomical characteristics.

**Table 1:** Patient characteristics at baseline of patients with (functional) univentricular heart disease after Fontan palliation who underwent percutaneous closure of at least one veno-venous collateral.

Patient characteristics at baseline	Total cohort (n = 49)	Patients without percutaneous closure procedure (n = 36)	Patients who underwent percutaneous closure procedure at adult age (n = 13)	p-value for the comparison between groups
Age, years (mean ± SD)	24 ± 6	23 ± 6	24 ± 4	0.232
Female	27 (55 %)	17 (47 %)	10 (77 %)	0.065
BSA, m^2^ (mean ± SD)	1.78 ± 0.2	1.78 ± 0.2	1.74 ± 0.3	0.880
BMI, kg/m^2^ (mean ± SD)	23 ± 4	22 ± 3	25 ± 5	0.140
**Anatomy and medical history**				
Diagnosis at birth				0.085
AVSD	5 (10 %)	5 (14 %)	0 (0 %)	
ccTGA	5 (10 %)	4 (11 %)	1 (8 %)	
Criss cross heart	1 (2 %)	0 (0 %)	1 (8 %)	
Double outlet RV	7 (14 %)	5 (14 %)	2 (15 %)	
Double inlet LV	7 (14 %)	5 (14 %)	2 (15 %)	
Hypoplastic LV	9 (18 %)	9 (25 %)	0 (0 %)	
Mitral atresia	1 (2 %)	1 (3 %)	0 (0 %)	
Pulmonary stenosis/atresia	5 (10 %)	2 (6 %)	3 (23 %)	
Truncus arteriosus	1 (2 %)	1 (3 %)	0 (0 %)	
Tricuspid atresia	8 (16 %)	4 (11 %)	4 (31 %)	
Systemic left ventricle	24 (49 %)	14 (39 %)	10 (77 %)	0.060
Previous Blalock-Taussig Shunt	21 (43 %)	15 (42 %)	7 (54 %)	0.779
Fontan type				0.772
Atriopulmonary connection	4 (8 %)	3 (8 %)	1 (8 %)	
Lateral tunnel	5 (10 %)	3 (8 %)	2 (15 %)	
Extracardiac conduit	40 (82 %)	30 (83 %)	10 (77 %)	
Fontan conduit size, mm (median [Q1-Q3])	16 [16-16]	16 [16-16]	16 [16–20]	0.261
History of atrial arrhythmia	11 (22 %)	7 (19 %)	4 (31 %)	0.402
History of FALD	14 (29 %)	7 (19 %)	7 (54 %)	**0.019**
History of PLE	1 (2 %)	0 (0 %)	1 (8 %)	0.093

Data is shown as n (% of the cohort) unless indicated otherwise. Bold is statistically significant.

AVSD: atrioventricular septal defect, BMI: body mass index, BSA: body surface area, ccTGA: congenitally corrected transposition of the great arteries, FALD: Fontan-associated liver disease, LV: left ventricle, PLE: protein losing enteropathy, Q: quartile, RV: right ventricle, SD: standard deviation, VSD: ventricular septal defect.

Of the cohort who underwent a percutaneous closure procedure (n = 13), 77 % was female. The mean age at percutaneous closure procedure was 24 ± 4 years, median age at Fontan operation was 4 [2.5–6] years. Ten patients (77 %) had a systemic left ventricle, and 10 (77 %) had an extracardiac Fontan conduit with a median Fontan conduit size of 16 [16–20] mm. Seven patients (54 %) had Fontan-associated liver disease, one patient (8 %) had a history of protein losing enteropathy. Systolic function of the systemic ventricle on transthoracic echocardiography was preserved in 6 patients (46 %), and mean ejection fraction on magnetic resonance imaging was 46 ± 7 %. None of the patients had hemodynamically significant atrioventricular (AV)-valve regurgitation. Haemoglobin levels, platelet counts, renal function and NT-proBNP were all within normal reference range, and patients had a low (non-invasive) liver fibrosis score. The use of heart failure medication in this cohort was low (1 patient, 8 %). Detailed information on anatomy and medical history is shown in Table 1, Table 2.

**Table 2:** Functional, biochemical, and pharmacological data at baseline of patients with (functional) univentricular heart disease after Fontan palliation who underwent percutaneous closure of at least one veno-venous collateral.

Cardiac imaging	
Global systemic ventricle function on TTE	
Good	6 (46 %)
Mildly reduced	7 (54 %)
Systemic ventricle ejection fraction on MRI, % (mean ± SD)	46 ± 7
AV-valve regurgitation on TTE	
Grade 1 or less	10 (77 %)
Grade 2	3 (23 %)
Laboratory values (mean ± SD)	
Hb, mmol/L	9.1 ± 0.9
Ht, L/L	0.443 ± 0.03
Platelet count, 10^9^/L	201 ± 63
Sodium, mmol/L	140 ± 2
Potassium, mmol/L	4.2 ± 0.3
Creatinine, mmol/L	68 ± 19
eGFR, mL/min/1.73m^2^	112 ± 25
Albumin, g/L	46 ± 6
NT-proBNP, ng/L	149 ± 99
ASAT, U/L	32 ± 7
ALAT, U/L	39 ± 11
AF, U/L	104 ± 70
Gamma GT, U/L	91 ± 46
Liver fibrosis scores (mean ± SD)	
APRI^a^	0.55 ± 0.2
FIB-4 Index^b^	0.68 ± 0.2
Pharmacological therapy	
Vitamin K antagonist	9 (69 %)
Acetylsalicylic acid	4 (31 %)
Beta blocker	1 (8 %)
ACEi	1 (8 %)
MRA	1 (8 %)
Diuretics	1 (8 %)

Data is shown as n (%) unless indicated otherwise.

ACEi: angiotensin-converting enzyme-inhibitor, AF: alkaline phosphatase, ALAT: alanine transaminase, APRI: ASAT to platelet ratio index, ASAT: aspartase aminotransferase, AV: atrioventricular, eGFR: estimated glomerular filtration rate, FIB-4: Fibrosis-4, Gamma GT: gamma glutamyltransferase, Hb: haemoglobin, Ht: haematocrit, MRA: mineralocorticoid receptor antagonist, MRI: magnetic resonance imaging, NT-proBNP: N-terminal-pro hormone brain natriuretic peptide, Q: quartile, SD: standard deviation, TTE: transthoracic echocardiography.

a APRI threshold of 0.7: significant fibrosis [13].

b FIB-4 Index <1.45: 0–1 fibrosis stage [14].

### 3.2 Percutaneous closure procedure

The procedure was performed using femoral access under fluoroscopic guidance in the majority of patients (12 patients, 92 %), in one patient jugular vein access was used. The catheterization was performed under local anaesthesia in 12 patients (92 %) and conscious sedation in one patient (8 %). Venous filling pressures were low, Table 3, and no Fontan pathway obstruction was observed.

**Table 3:** Invasive hemodynamic measurements and anatomical details during catheterization.

Catheterization findings (n = 13 patients)	
Pressures, mmHg	
Vena cava inferior	10 ± 4
Fontan conduit	10 ± 4
Glenn	10 ± 4
Right pulmonary artery	9 ± 4
Left pulmonary artery	10 ± 4
Systolic aorta (non-invasive)	123 ± 11
Diastolic aorta (non-invasive)	73 ± 11
Vena cava inferior and Fontan conduit gradient	0.09 ± 0.5
**Veno-venous collaterals (n = 25)**	
Location (n, %)	
Tributary of the vena cava superior to pulmonary veins	8 (32 %)
Tributary of the vena cava superior to atrium	3 (12 %)
Tributary of the vena cava superior to coronary sinus	3 (12 %)
Tributary of the vena cava inferior to pulmonary veins	11 (44 %)
VVC per patient, median [Q1-Q3]	2 [1.5–2]
**Treatment of veno-venous collaterals**	
Conservative	2 (8 %)
Closure	23 (92 %)
Coils	16 (70 %)
Amplatzer device	7 (30 %)

Data is shown as mean ± standard deviation unless indicated otherwise.

Q: quartile, VVC: Veno-venous collaterals.

**Fig. 2:**
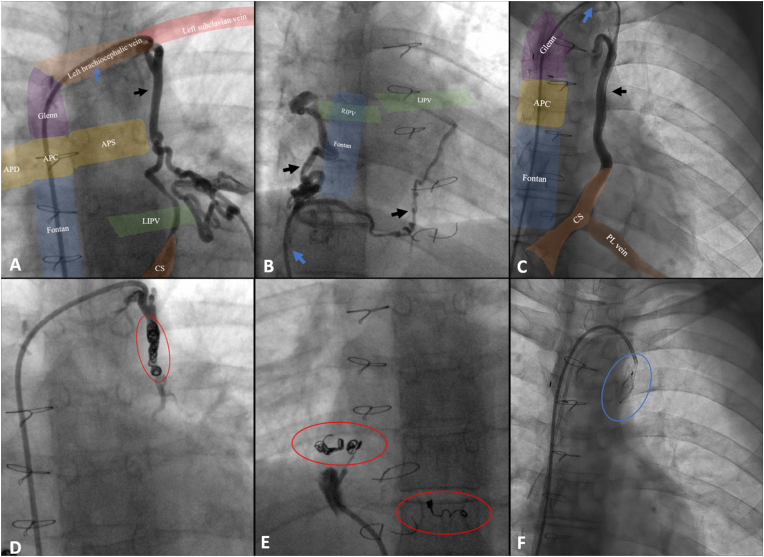
Angiographic projection showing: (A) collateral flow from left subclavian vein to left inferior pulmonary venous return. (B) collateral flow (black arrow) from inferior vena cava to right and left inferior pulmonary venous return. (C) collateral flow from left subclavian vein to coronary sinus. (D, E) after coiling (red circle) there is reduced distal contrast opacification and (F) after closure with an Amplatzer Vascular plug (blue circle) APC: pulmonary trunk; APD: right pulmonary artery, APS: left pulmonary artery, CS: coronary sinus, LIPV: left inferior pulmonary vein, PL: posterolateral, RIPV: right inferior pulmonary vein. Black arrow: collateral, blue arrow: catheter, blue circle: Amplatzer Vascular plug II (*Abbott*), red circle: vortX pushable coils (*Boston Scientific*).

**Fig. 3:**
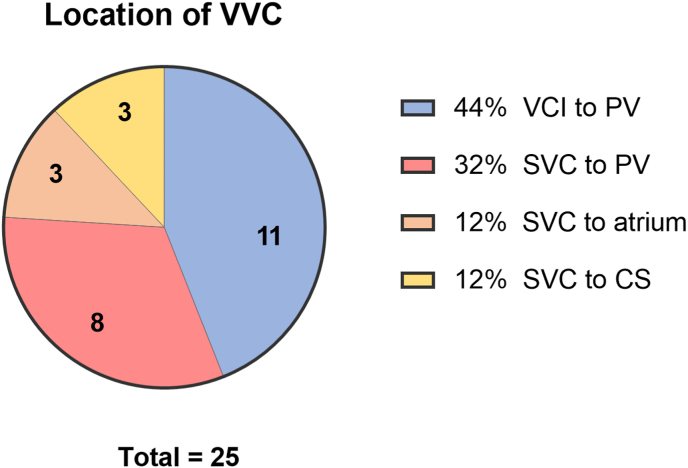
Pie chart visualizing the origin and insertion of the veno-venous collaterals. CS: coronary sinus, PV: pulmonary veins, VCI: tributaries of the vena cava inferior, SVC: tributaries of the vena cava superior, VVC: veno-venous collaterals.

A total of 25 VVC were identified in 13 patients, Table 3, Fig. 2. The VVC originated from the tributaries of the vena cava inferior to pulmonary veins in 11 VVC (11/25: 44 %), the tributaries of the vena cava superior and connected to pulmonary veins (8 VVC,8/25: 32 %), the tributaries of the vena cava superior to systemic atrium (3 VVC, 3/25: 12 %), or the tributaries of the vena cava superior to coronary sinus (3 VVC, 3/25: 12 %), Fig. 3. Seventeen VVC (68 %) were classified as large collaterals, of which 79 % (11/14) were tributaries of the vena cava superior versus 55 % (6/11) were tributaries of the vena cava inferior.

A total of 23 VVC were closed, using coils in 16 VVC (70 %) and Amplatzer device (Vascular plug) in 7 (30 %). A mean of 2.9 ± 1.8 coils was used per VVC. In all patients, at least one VVC was closed. The reason for not closing the VVC (n = 2) was in both cases a very small diameter of the VVC with a sharp offset angle, hampering stable selective cannulation. The result was satisfactory in all patients, without significant residual shunt at angiography. No periprocedural complications occurred, patients were discharged the same or following day after the procedure.

### 3.3 Clinical outcomes

A follow-up moment 6 months after closing procedure was available in 11 patients with a median follow-up of 13 [10–24] months. One patient had concurring medical problems in a different hospital and was unable to follow-up at our tertiary centre in the time frame of this study. The other patient had sarcopenia and strongly reduced intake for which the patient was under intensive supervision of the dietologist and was unable to follow the structured follow-up, resulting in a lack of follow-up moment within the time frame of this study. While this did result in loss to follow-up of two patients within the study population, it is, unfortunately, reflective of the multiorgan impairment of Fontan patients.

**Fig. 4:**
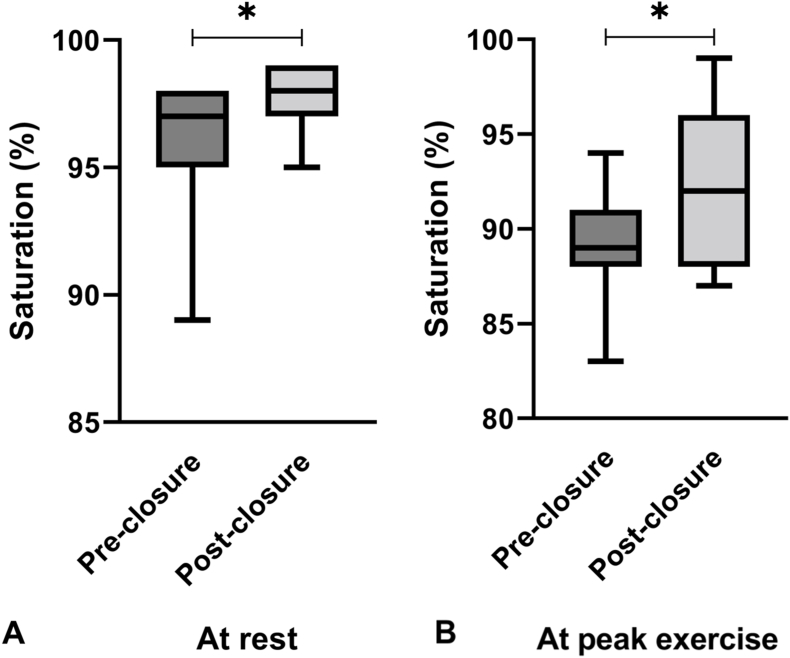
Box and whisker plot showing the median peripheral oxygen saturation at ambient air (horizontal line) with 25th and 75th percentiles (box) and lower and upper extremes (whiskers) pre- and post-closure of the veno-venous collaterals (A) at rest, (B) at peak exercise. * statistically significant (p < 0.05).

No post procedural complications were documented. Nine patients (82 %) had self-reported reduction of symptoms. Mean peripheral saturation at rest and during maximal exercise improved significantly (96 ± 3 to 98 ± 1 %, p = 0.040 and 89 ± 3 to 93 ± 5 %, p = 0.024, respectively), Fig. 4, Table 4. Clinical, echocardiographic, and laboratory values and exercise capacity remained unchanged**,** Table 4**.**

**Table 4:** Clinical outcomes before and after coiling in the patients with (functional) univentricular heart disease after Fontan palliation who had follow-up at least 6 months after the catheterization.

Patients after Fontan palliation with follow-up at least 6 months after catheterization (n = 11)	Pre-coiling	Post-coiling	p-value
Clinical and echocardiographic values			
Weight, kg	69 ± 4	73 ± 4	0.052
Systemic ventricle function (n, %)			0.500
Good	5 (46 %)	3 (27 %)	
Mildly reduced	6 (54 %)	8 (73 %)	
AV-valve regurgitation (n, %)			1.000
Grade 1 or less	9 (82 %)	9 (82 %)	
Grade 2	2 (18 %)	2 (18 %)	
**Laboratory values**			
Hb, mmol/L	9.1 ± 1.0	9.1 ± 0.9	0.937
Ht, L/L	0.446 ± 0.03	0.446 ± 0.04	0.992
eGFR, mL/min/1.73m^2^	106 ± 25	109 ± 30	0.524
ASAT, U/L	33 ± 7	33 ± 10	0.888
ALAT, U/L	43 ± 12	44 ± 18	0.676
Gamma GT, U/L	115 ± 36	128 ± 57	0.174
NT-proBNP, ng/L	175 ± 118	199 ± 104	0.093
**Bicycle ergometry**			
Saturation at rest, %	96 ± 3	98 ± 1	**0.040**
Saturation at peak exercise, %	89 ± 3	93 ± 5	**0.024**
Delta saturation rest and peak exercise, %	7 ± 4	5 ± 4	0.144
Exercise capacity, watt	135 ± 40	136 ± 39	0.879
Exercise capacity (watt), % of predicted	76 ± 14	76 ± 19	0.939
VO_2_max, mL/kg/min	22.1 ± 5.4	21.1 ± 5.1	0.356
Percent of predicted VO_2_max, %	56 ± 11	56 ± 13	0.833

Data is shown as mean ± standard deviation unless indicated otherwise. Bold is significantly different (p < 0.05).

ALAT: alanine transaminase, ASAT: aspartase aminotransferase, AV: atrioventricular, eGFR: estimated glomerular filtration rate, Gamma GT: gamma glutamyltransferase, Hb: haemoglobin, Ht: haematocrit, NT-proBNP: N-terminal-pro hormone brain natriuretic peptide.

### 3.4 Literature review on prevalence of veno-venous collaterals in Fontan patients

A total of 121 articles were found with the search strategy after removal of duplicates. After further investigation 18 articles were selected for this systematic review. Fourteen articles were reviewed for prevalence and associated factors with the occurrence of VVC and 13 articles for closure strategy and outcomes (Fig. 1).

#### 3.4.1 Prevalence of veno-venous collaterals in Fontan patients

Fourteen studies in patients with a Fontan circulation on VVC prevalence and factors associated with VVC were identified [[10], [11], [12],[18], [19], [20], [21], [22], [23], [24], [25], [26], [27], [28]]. An overview of the studies of VVC in these patients is shown in Table 5, Table 6. A total of 13 studies described the prevalence of VVC in their cohort [10,11,[18], [19], [20], [21], [22], [23], [24], [25], [26], [27], [28]]. The prevalence in these studies ranged from 4 % to 80 % in diverse types of study populations. In the adult only cohort, the prevalence was 58 % [11].

**Table 5:** Epidemiological, anatomical, and hemodynamic details of all included studies on VVC in patients with (functional) univentricular heart disease after Fontan palliation.

	Author and year	Type of study	Population screened	Primary aim	Number of Fontan patients	Age	Sex, female	Age at Fontan operation	Systemic left ventricle	Type of Fontan	Invasive Fontan or right sided pressures	Oxygen saturation at rest
1.	Heinemann et al., 2001 [19] * †	Retrospective cohort	All patients with Fontan-type operation	Assess incidence and predilection sites of VVC as well was therapeutic options	62	Not specified	Not specified	5.9 years	For the 7 Fontan patients with VVC: 5 (71 %)	For the 7 Fontan patients with VVC: Lateral tunnel 4 (57 %), AP 1 (14 %), other: 2 (29 %)	Not specified	For the 7 Fontan patients with VVC: 83 ± 4 %
2.	Kaulitz et al., 2002 [20] * †	Retrospective cohort	All Fontan patients, excluding patients with early death	Assess the type and incidence of hemodynamic and electrophysiological abnormalities requiring surgical or interventional procedures during long-term follow-up after Fontan-type operations	88	At evaluation: 15 ± 6 years	Not specified	6.1 ± 5 years	Not specified	TCPC: 72 (82 %), AP anastomosis: 16 (18 %)	Right atrial pressure 11.7 ± 3 mmHg	93 ± 4 %
3.	Sugiyama et al., 2003 [12] * †	Retrospective cohort	All Fontan patients who underwent interventional procedure for VVC	Establish the mechanisms underscoring the development of VVC and their optimal treatment	50	At procedure: 6 ± 4 years	22 (44 %)	3.6 ± 2.1 years	32 (64 %)	Extracardiac conduit: 16 (32 %), lateral tunnel: 29 (58 %), AP anastomosis: 2 (4 %), other: 3 (6 %)	mPAP 12.2 ± 2.7 mmHg	88 ± 6 %
4.	Girisch et al., 2005 [32] †	Case report (n = 2)	Not specified	Describe the interventional treatment in two Fontan patients with re-opened left and right superior vena cava	2	At closure procedure: 19 and 21 years	Not specified	14 and 11 years	2 (100 %)	TCPC: 1 (50 %), AP 1 (50 %)	mPAP 11 and 12 mmHg	89 ± 4 %
5.	Vogt et al., 2007 [28] * †	Retrospective cohort	All Fontan patients who underwent cardiac catheterization before BCPS, between BCPS and Fontan, and after Fontan operation	Characterize somatic growth and define associated factors (hemodynamic and collateral vessels)	126	Not specified	44 (35 %)	2.7 [1.6–11.2]	80 (63 %)	Extracardiac conduit: 89 (72 %), lateral tunnel: 36 (28 %), AP anastomosis: 1 (1 %)	mPAP 12 ± 3 mmHg	92 ± 6 %
6.	Masura et al., 2008 [23] * †	Retrospective cohort	All Fontan patients who underwent cardiac catheterization for cyanosis (saturation <90 %)	Report diagnosis and percutaneous management of cyanosis in Fontan patients.	41	Median at catheterization: 8 years	Not specified	Age 8 [2.5–26]	Not specified	Extracardiac conduit: 19 (46 %), lateral tunnel 22 (54 %)	Mean systemic venous pressure for patients with VVC: 7.8 ± 2.4 mmHg	Patients with VVC: 76 ± 3 %
7.	Schwartz et al., 2010 [31] †	Retrospective cohort	All congenital heart disease patients who underwent a vascular occlusion procedure	Describe the experience with plug 1 and 2 in patients with congenital cardiovascular disease	7 where VVC were occluded	Cohort venous collaterals (including venous-arterial): 12 [0.8–47] years	Not specified	All congenital patients: 2.0 years	Not specified	Not specified	Not specified	Not specified
8.	Goldstein et al., 2012 [30] †	Retrospective cohort	All congenital heart disease patients who underwent attempted vascular occlusion procedure	Describe the experience with the hydrocoil for vascular occlusion in congenital cardiovascular disease	3 Fontan patients	Of the 3 Fontan patients: 11 ± 8 years	Of the 3 Fontan patients: 2 (67 %)	Not specified	Of the 3 Fontan patients: 2 (67 %)	Not specified	Not specified	Not specified
9.	Lluri et al., 2015 [11] * †	Retrospective cohort	All adult Fontan patients who underwent cardiac catheterization	Characterize the anatomic and clinical features of VVC in adult Fontan patients	66	At catheterization: 29 ± 8 years	42 (66 %)	11.5 ± 8.7 years	46 (70 %)	Extracardiac conduit: 20 (30 %), lateral tunnel 25 (38 %), AP anastomosis: 21 (32 %)	Mean Fontan pressure in the patients with intervention: 14.7 ± 2.7 mmHg	In the patients with intervention: 86 ± 6 %
10.	Poterucha et al., 2015 [10] * †	Retrospective cohort	All Fontan patients who underwent cardiac catheterization	Determine frequency of VVC and outcomes of closure	496	Of patients with diagnosis of VVC at catheterization: 26 ± 12 years	In the patients with VVC: 35 (49 %)	In the patients with VVC and embolization: 7 ± 7 years (without 9 ± 12 years)	In the patients with VVC: 47 (66 %)	In the patients with VVC: Extracardiac conduit: 11 (15 %), lateral tunnel 25 (35 %), AP anastomosis: 36 (50 %)	In the patients with VVC and embolization: Mean Fontan pressure 18 ± 6 mmHg (without 16 ± 4 mmHg)	In the patients with VVC and embolization: 88 ± 7 % (without 90 ± 6 %)
11.	Lafuente et al., 2016 [22] * †	Retrospective cohort	All Fontan patients who underwent interventional catheterization procedures	Describe different interventional catheterizations	85	Not specified	Not specified	Not specified	Not specified	Extracardiac conduit: 78 (92 %), lateral tunnel 1 (1 %), AP anastomosis: 4 (5 %) Not defined: 2 (2 %)	Not specified	Not specified
12.	Evans et al., 2020 [18] *	Retrospective cohort	All living Fontan patients with cardiac catheterization and transvenous liver biopsy	Evaluate the relationship between post‐Fontan VVC and a range of hepatic fibrosis scores and the rate of hepatic fibrosis progression	164	At catheterization and biopsy: 21 ± 9 years	58 (35 %)	In the patients with VVC: 3.9 ± 1 years (without 3.5 ± 1 years)	89 (54 %)	Extracardiac conduit: 119 (73 %), lateral tunnel 33 (20 %), AP anastomosis: 12 (7 %)	In the patients with VVC: vena cava inferior pressure 13 ± 2 mmHg (without 13 ± 2 mmHg)	In the patients with VVC: 91 ± 4 % (without 93 ± 3 %)
13.	Komori et al., 2020 [21] *	Retrospective cohort	All Fontan patients who underwent cardiac catheterization, spirometry, and exercise test 10 years after Fontan operation	Evaluate the impact of PNP on respiratory function, hemodynamic, and exercise capacity 10 years after Fontan procedure	160	At catheterization: 14 ± 3 years	Not specified	In patients without PNP 2.4 ± 1.8 years (recovered: 1.5 ± 0.6 years, persistent: 2.9 ± 2.5 years)	Not specified	Extracardiac conduit: 160 (100 %)	In patients without PNP: mPAP 9.3 ± 2.1 mmHg (recovered: 9.4 ± 1.6 mmHg, persistent: 11.5 ± 2.8 mmHg)	In patients without PNP: 95 ± 3 % (recovered: 95 ± 2 %, persistent: 92 ± 5 %)
14.	Ozawa et al., 2021 [25] *	Retrospective cohort	All Fontan patients	Assess long-term outcomes after fenestration closure in patient at risk for Fontan failure	117	Not specified	Not specified	In patients without initial fenestration 3.3 ± 4.5 years (F-SR: 9.0 ± 12.9 years, F-HR: 7.2 ± 5.8 years)	In patients without initial fenestration 37 (42 %) (F-SR: 4 (33 %), F-HR: 7 (44 %))	Extracardiac conduit: 114 (97 %), AP anastomosis: 3 (3 %)	Not specified	Not specified
15.	Raimondi et al., 2021 [26] *	Retrospective cohort	All Fontan patients who underwent cardiac MRI with 4D flow acquisition	Assess relative reliability of flow measurements and observe frequency of venous shunting on 4D flow MRI	75 (42 adult Fontan patients)	At cardiac MRI: 20 [5–58] years	46 (61 %)	4 [1–19] years	39 (52 %)	Extracardiac conduit: 47 (63 %), lateral tunnel 15 (20 %), AP anastomosis: 13 (17 %)	Not specified	Not specified
16.	Schafstedde et al., 2021 [27] * †	Retrospective cohort	All Fontan patients with at least 1 follow-up examination after Fontan operation	Describe the prevalence and clinical outcome of cyanosis after Fontan operation	331	Not specified	161 (49 %)	3.9 [2.8–6.3]	232 (70 %)	Extracardiac conduit: 244 (74 %), lateral tunnel 67 (20 %), AP anastomosis: 20 (6 %)	Post-Fontan mPAP: 12.0 [9.3–14.0] mmHg	Post-Fontan 92 [88–94]
17.	Ohuchi et al., 2022 [24] *	Retrospective cohort	All Fontan patients who survived at least 6 months after the operation and had PAVF evaluation	Assess incidence of PAVF and association with Fontan pathophysiology	391	Not specified	161 (41 %)	In patients without PAVF: 4 ± 5 years (diffuse PAVF: 5 ± 6 years, discrete PAVF: 8 ± 5 years)	155 (40 %)	Extracardiac conduit: 153 (40 %), lateral tunnel 143 (37 %), AP anastomosis: 4 (1 %) Not defined: 91 (23 %)	At time of detection PAVF. In patients without PAVF CVP: 10 ± 3 mmHg (diffuse PAVF: 12 ± 3 mmHg, discrete PAVF: 10 ± 3 mmHg)	At time of detection PAVF. In patients without PAVF: 94 ± 3 % (diffuse PAVF: 92 ± 4 %, discrete PAVF: 91 ± 1 %)
18.	Baba et al., 2022 [29] †	Retrospective cohort	All patients who underwent transcatheter vascular embolization with 0.035-inch hydrogel coils	Describe the experience with transcatheter vascular occlusion using 0.035-inch hydrogel expandable coils	15	Of all 20 patients described: 5.1 [0.05–26.0] years	Of all 20 patients described: 9 (45 %)	Not specified	4 (27 %)	Not specified	Not specified	Not specified

Data is displayed as mean ± standard deviation, median [range] or number (percentage). Data is shown for all (screened) Fontan patients, unless explicitly noted otherwise. * studies identified for prevalence and associated factors of VVC, † studies identified for percutaneous closure strategy and outcome of VVC.

AP: atrio-pulmonary, BCPS: bidirectional cavopulmonary shunt, CVP: central venous pressure, Fontan patients: patients with (functional) univentricular heart disease after Fontan palliation, F-HR: high risk Fontan patients with initial fenestration (at pre-Fontan evaluation a mean pulmonary arterial pressure ≥15 mmHg and/or preoperative systemic atrioventricular valve regurgitation ≥ moderate), F-SR: standard risk Fontan patients with initial fenestration, mPAP: mean pulmonary artery pressure, MRI: magnetic resonance imaging, PAVF: pulmonary arteriovenous fistulae, PNP: phrenic nerve palsy, SD: standard deviation; TCPC: total cavopulmonary connection, VVC: veno-venous collaterals, 4D: four-dimensional.

**Table 6:** Prevalence, functional aspects, factors, and findings of VVC in patients with (functional) univentricular heart disease after Fontan palliation.

	Author and year	Prevalence of VVC (n, %)	Location of VVC	Factors associated with VVC	Main findings/conclusion related to VVC
1.	Heinemann et al., 2001 [19]	7/62: 11 %	50 % above the diaphragm, 38 % below the diaphragm, 12 % cardiac. 75 % connecting to the functional left atrium, 25 % to the pulmonary veins.	NOT age at Fontan	Progressive cyanosis is an indication for early re-evaluation
2.	Kaulitz et al., 2002 [20]	11/88: 13 %	All above the diaphragm connecting to pulmonary veins or left atrium.	NOT anatomy or postoperative hemodynamics	Collaterals have to be suspected in patients with increasing or persisting cyanosis
3.	Sugiyama et al., 2003 [12]	Not specified	71 % above the diaphragm, 29 % below the diaphragm, mostly connecting to the atrium or pulmonary veins	Larger collaterals were found in patients with a longer interval between Fontan operation and catheterization, with higher mean pulmonary arterial pressures	Venous collateral channels are common in patients suffering progressive cyanosis in the setting of the Fontan circulation. The collaterals increase in size with time, and are associated with higher pulmonary arterial pressures
4.	Vogt et al., 2007 [28]	49/126 : 39 %	Not specified	Both post BCPS and post-Fontan: presence was associated with lower arterial oxygen saturation, higher mean pulmonary artery pressure, higher right atrial pressure, and higher end diastolic pressure in the dominant ventricle. The presence of 2 or more active venous collaterals was associated with impaired growth	Incidence of VVC increased per stage of correction for univentricular hearts. The presence of venous collaterals is likely associated with impaired growth secondary to hemodynamic impairment and hypoxemia. In patients that have venous collaterals embolized, growth potential might be restored
5.	Masura et al., 2008 [23]	3/41: 7 %	100 % above the diaphragm, insertion not specified.	Not specified	Progressive decline of percutaneous oxygen saturation during follow-up after Fontan is suggestive of development of venous collaterals or pulmonary arteriovenous malformations
6.	Lluri et al., 2015 [11]	38/66: 58 %	85 % above the diaphragm, 15 % below the diaphragm. All connecting to pulmonary veins	NOT duration of Fontan, systemic venous pressure or patient age	Systemic to pulmonary venous collaterals are commonly found in this population but their presence is not predicted by duration of Fontan, systemic venous pressure, or patient age
7.	Poterucha et al., 2015 [10]	72/496: 15 %	80 % above the diaphragm, 20 % below the diaphragm. 65 % connecting to pulmonary veins, 18 % atrium, 17 % coronary sinus.	Not specified	Embolization of VVC in patients after Fontan should be evaluated very carefully in patients with AP-type Fontan, heterotaxy, and those with Fontan pressure above 18 mm Hg
8.	Lafuente et al., 2016 [22]	28/85: 33 %	Not specified	Not specified	Interventional catheterization is essential in management of Fontan patients
9.	Evans et al., 2020 [18]	101/164: 62 %	Not specified	Higher liver fibrosis score, rate fibrosis progression, and elastography values, and lower saturation	The presence of angiographically demonstrated VVC was associated with statistically, significantly more advanced liver fibrosis than those without collaterals
10.	Komori et al., 2020 [21]	7/160: 4 %	Not specified	Recovered or persistent phrenic nerve palsy and diaphragmatic plication	Diaphragmatic plication should be avoided as much as possible to prevent later development of VVC
11.	Ozawa et al., 2021 [25]	15/22: 68 % in Fontan patients with an initial fenestration at 15 years after Fontan operation	Not specified	An initial fenestration during Fontan procedure and at pre-Fontan evaluation a mean pulmonary arterial pressure ≥15 mmHg and/or preoperative systemic atrioventricular valve regurgitation ≥ moderate	Cyanosis remained after fenestration closure in high-risk Fontan patients due to natural transformation to VVC, which resulted in elevation of pulmonary vascular resistance, low cardiac index, and deterioration of exercise intolerance
12.	Raimondi et al., 2021 [26]	30/75: 40 %	Origin not specified. Most frequently connected to right pulmonary veins or atrium	Not specified	The severity of veno-venous shunting appeared to contribute substantively to high cardiac output state
13.	Schafstedde et al., 2021 [27]	86/178: 48 % in patients who underwent catheterization for decreasing saturation	Not specified	Lower saturation	Persisting or reoccurring cyanosis during long-term follow-up after Fontan operation may represent an important indicator of impaired Fontan hemodynamics that should prompt adequate diagnostic assessment and optimization of treatment
14.	Ohuchi et al., 2022 [24]	74/100: 74 %, 97/128: 80 %, 97/153: 65 %, 82/126: 65 %, 50/78: 65 %, 26/48: 54 % at 1, 5, 10, 15, 20 and 25 years after Fontan operation respectively	Not specified	PAVF	A high prevalence of VVC was found and VVC often coexisted with PAVF

BCPS: bidirectional cavopulmonary shunt, Fontan patients: patients with (functional) univentricular heart disease after Fontan palliation, MRI: magnetic resonance imaging, PAVF: pulmonary arteriovenous fistulae, SD: standard deviation; VVC: veno-venous collaterals, 4D: four-dimensional.

The common presentation of the VVC was a reduced oxygen saturation level at rest. Factors statistically significantly associated with the prevalence of VVC were liver fibrosis, rate of fibrosis progression, elastography values, the presence of pulmonary arteriovenous fistulae, recovered or persistent phrenic nerve palsy, diaphragmatic plication, high mean pulmonary artery pressure, high right atrial pressure, high end diastolic pressure in the dominant ventricle, and/or high risk Fontan patients who received an initial fenestration at Fontan operation (high risk was defined as mean pulmonary arterial pressure ≥15 mmHg and/or preoperative systemic AV-valve regurgitation ≥ moderate at the evaluation before the Fontan completion). Larger collaterals were found in patients with a longer interval between Fontan operation and catheterization, as well as in those with higher mean pulmonary arterial pressures. Factors that were found not to be statistically significantly associated with VVC were age at Fontan operation, underlying cardiac anatomy, postoperative hemodynamics, systemic venous pressures, Fontan duration (the period after Fontan completion) and/or patient age.

### 3.5 Closure strategy of veno-venous collaterals in Fontan patients

A total of 13 studies were identified reporting on feasibility of closing procedure and complication reports. An overview of the percutaneous closure strategy, and outcomes of VVC in Fontan patients is shown in Table 5, Table 7. In all studies the closure technique was technical feasible without the occurrence of complications, comparable to our experience [[10], [11], [12],^19^,^20^,[27], [28], [29], [30], [31], [32]]. Out of the 268 patients undergoing percutaneous closure of the VVC in all the studies combined, the closure technique used was coiling in 208 patients (77 %), plugs in 24 (9 %), Rashkind occluders in 5 (2 %), and a ventricular septum defect occluders in 2 (1 %), and in 29 patients (11 %) the technique was not specified.

**Table 7:** Percutaneous closure strategy and outcomes of VVC in patients with (functional) univentricular heart disease after Fontan palliation.

	Author and year	Presentation	Closure technique	Patients with VVC treated (n, %)	Short term outcome	Complications	Follow-up	Main findings/conclusion in relation to closure strategy
1.	Heinemann et al., 2001 [19]	Progressive desaturation or detection during standard follow-up procedure	Coils	3/9	Increase in saturation (rest) post procedure	None	No	Catheter intervention plays the leading role for occluding unwanted vascular pathways, because it is much less invasive than reoperation and can be combined with the diagnostic procedure.
2.	Kaulitz et al., 2002 [20]	Arterial saturation <85 %	Catheter closure not specified	2/11	Increase in saturation (rest) post procedure	None	No	None
3.	Sugiyama et al., 2003 [12]	Desaturation	Coils (90 %) and Rashkind occluder (10 %)	50/50 (24/50 had simultaneous fenestration closure)	Increase in saturation (independent of fenestration closure)	None	No	Transcatheter treatment is feasible, and results in resolution of cyanosis
4.	Girisch et al., 2005 [32]	Desaturation	Amplatzer VSD Occluder	2/2	Increase in saturation (rest)	None	Persisted increase in saturation after 2 and 3 years, respectively	The closure of recanalized bilateral superior caval veins after Fontan procedures is possible without technical problems by means of the Amplatzer® muscular VSD Occluder.
5.	Vogt et al., 2007 [28]	Routine	Coils (embolization)	41/49	No	None	No	In patients that have VVC embolized, growth potential might be restored
6.	Masura et al., 2008 [23]	Cyanosis	Plugs (Amplatzer vascular plugs)	3/3 (2/3 had simultaneous fenestration closure)	Increase in systemic venous pressure (7.8 ± 2.4 to 9.8 ± 2.3)	None	Increase in saturation after median of 14 months	Progressive decline of percutaneous oxygen saturation during follow-up after Fontan completion is suggestive of development of venous collaterals or pulmonary arteriovenous malformations. Superfluous communications are amenable to percutaneous closure using various types of Amplatzer occluders.
7.	Schwartz et al., 2010 [31]	Not specified	Plugs (Amplatzer vascular plug)	7/7	No	None	No	Amplatzer vascular plugs 1 and 2 are versatile, and highly effective devices for occlusion of a variety of lesions in congenital heart disease.
8.	Goldstein et al., 2012 [30]	Cyanosis	Coils	3/3	No	None	No	Hydrocoils are safe and effective devices for vascular occlusion in a variety of circumstances routinely encountered in the treatment of patients with congenital cardiovascular disease.
9.	Lluri et al., 2015 [11]	Not specified	Not specified	27/38	Increase in saturation at 6 months post-procedure	None	60 % had follow-up >24 months: small rise from 6 to 24 months (still significantly higher than baseline)	Interventional closure of veno-venous collaterals in adults is associated with intermediate and long-term improvements in systemic oxygen saturation with a low risk of complications
10.	Poterucha et al., 2015 [10]	Desaturation, pre-heart transplantation, clinical evaluation	Coils (52 %) and plug (48 %)	31/72	No increase in saturation	None	Decreased 5-year survival. 2.5 years after closure: no change in saturation and haemoglobin. 5 years after closure: decreased cardiac index.	Embolization of VVC in patients after Fontan should be evaluated very carefully in patients with AP-type Fontan, heterotaxy, and those with Fontan pressure above 18 mm Hg
11.	Lafuente et al., 2016 [22]	Cyanosis	Coils (93 %) and plug (7 %)	28/85	No	None	No	Although VVC embolization improves oxygen saturation, we need to continue the evaluation in the long-term follow-up
12.	Schafstedde et al., 2021 [27]	Desaturation	Coils (embolization)	67/86	Increase in saturation	None	No	Individual haemodynamic findings and patients' characteristics will have to be considered for the management of residual right-to-left shunt by patent fenestrations or VVC, especially in the context of Fontan failure, as these may fundamentally differ.
13.	Baba et al., 2022 [29]	Not specified	Coils	7/7	No	None	No	The 0.035-inch hydrogel expandable coils are effective and safe in patients with congenital heart disease and vascular anomalies such as APC, VVC, and PAVM.

AP: atrio-pulmonary, APC: aortopulmonary collateral, Fontan patients: patients with (functional) univentricular heart disease after Fontan palliation, PAVM: pulmonary arteriovenous malformations, VVC: veno-venous collaterals, VSD: ventricular septal defect.

**Fig. 5:**
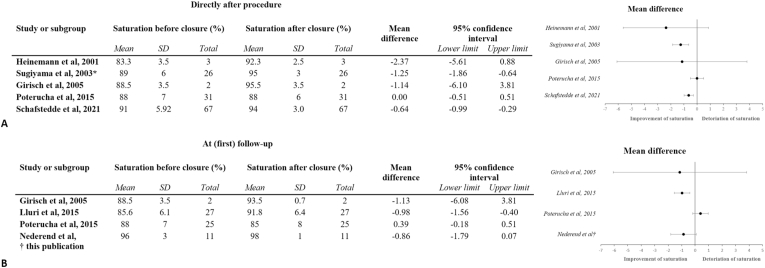
Forest plot for (A) oxygen saturation at rest directly after the procedure, and (B) peripheral saturation at rest at (first) follow-up SD: standard deviation. * patients without simultaneous fenestration closure.

A total of 8 studies reported either short-, medium- and/or long-term results, of which 7 were positive in terms of increase in oxygen saturation. Five studies found a direct increase in saturation post-closing procedure, of which one additionally reported that this persisted up to 2 and 3 years after the procedure [12,19,20,27,32]. One study found an increase in saturation 6 months post-procedure with a small decline in saturation again at 24 months of follow-up (yet still significantly higher than baseline) and an additional study reported a significant rise in saturation after a median of 14 months [11,23]. One study reported a negative finding, with no increase in saturation both post-procedurally and after 2 years, and additionally a decreased 5-year survival [10]. The mean difference in saturation for patients without simultaneous closure of fenestration is visually depicted in a Forest plot (Fig. 5).

#### 3.5.1 Gaps in literature

Only limited data was reported on long term clinical follow-up, symptoms, adult-only Fontan patients, influence of lifestyle and sex, and the specific driving factors for the development of VVC. No data was reported on follow-up of peripheral oxygen saturation at (peak) exercise.

## 4 Discussion

The main findings of this study are that (1) VVC in the adult Fontan population typically connect the tributaries of the vena cava inferior and/or superior with the pulmonary veins (2). Low Fontan pressures, preserved systemic ventricular function and “stable clinical” status do not exclude the presence of VVC (3). Percutaneous closure of VVC in this selected group of adult Fontan patients is technically feasible and safe and is associated with reduction of symptoms and a rise in peripheral oxygen saturation at rest and peak exercise (4). There are still several gaps in literature, including data on long term follow up in adult Fontan patients and driving factors for VVC development.

### 4.1 Prevalence of VVC

The prevalence of VVC reported in the literature, ranges from 4 % to up to 80 % of Fontan patients [10,11,[18], [19], [20], [21], [22], [23], [24], [25], [26], [27], [28]]. This is likely explained by the varying phenotypes (from “clinically stable” to “failing Fontan”) of the patients included and the screening strategy used (from incidental finding during follow-up to active screening for VVC). The studies with the lowest prevalence of VVC both studied cardiac catheterization data of a relatively young Fontan cohort [21,23].

### 4.2 Location of VVC

The substrate of the VVC is yet unknown. Their formation was initially thought to be related to elevated systemic venous pressures and decreased preload. However, the driving factors for VVC development are still a matter of debate, either recanalization of embryological structures or cyanosis-driven angiogenesis have been proposed [11,33,34]. The typically observed connection of the tributaries of the vena cava inferior and/or superior with the pulmonary veins, and especially the high prevalence of the recanalized left vena cava superior seems to support an embryological origin. During early embryology, when the central pulmonary venous return has not developed yet, extensive pulmonary to systemic venous connections exist in the splanchnic vascular pulmonary network [11,35]. During this initial peripheral drainage period, there is no open connection between the primitive atrium and the developing pulmonary veins. During the subsequent intermediate and central drainage, connections between the pulmonary and systemic veins will gradually obliterate and blood will find its definitive route to the left atrium via the lumenized anlage of the pulmonary vein – to later develop into the pulmonary veins [35]. It can therefore be hypothesized that in a patient with a Fontan circulation, recanalization of previously existing systemic-to-pulmonary vessels may occur, isolated or combined with a process of angiogenesis (remodelling and expansion) or even vasculogenesis (newly formed vessels) [36].

### 4.3 Factors associated with VVC in Fontan patients

The common characteristic of clinical presentation is progressive and/or exercise exacerbated cyanosis, which is a well-known marker that should prompt further evaluation [4,5]. The review of factors associated with VVC in the different studies provide insight into the mechanisms that might trigger VVC development in Fontan patients, such as advanced liver fibrosis, the presence of pulmonary arteriovenous fistulae, diaphragmatic plication, at least moderate systemic AV-valve regurgitation preoperatively, and higher mean pulmonary artery, right atrial or ventricular end diastolic pressure. Table 4 confirms that VVC can develop despite low right-sided pressures. Additionally, it is interesting that longer interval between Fontan operation and catheterization and higher mean pulmonary arterial pressures are associated with the size of the found collaterals, yet not with the development of VVC per se [12]. The development of VVC is not related to patient age, age at Fontan, post-operative (Fontan operation) hemodynamics or underlying anatomy [11,19,20]. There might be a role for invasive hemodynamic measurements during exercise to unmask and further unravel the complex hemodynamics, especially during exercise, and functional impairment in Fontan patients [37]. Evans et al. saw a relation between the presence of VVC and the severity of liver fibrosis (invasive measurements of liver fibrosis, rate of fibrosis progression, elastography values) [18]. In our cohort, the majority of patients had Fontan-associated liver disease, yet with low non-invasive fibrosis scores as assessed by the FIB-4 and the APRI. These scores were not developed and/or validated in the congenital heart disease population, and a study by Martin de Miguel et al. confirmed that the use of these scores in the Fontan population is poorly correlated with other markers of liver fibrosis, yet is associated with long-term all-cause mortality [38]. This supports that our cohort represents relatively “clinically stable” Fontan patients. Unfortunately, no invasive liver parameters were collected. It could be hypothesized that genetics potentially contribute to the occurrence and severity of VVC. Indeed, certain gene variants found in Fontan patients may render them more susceptible to specific long-term complications, such as the *BRAF* gene associated with Noonan spectrum disorders and protein-losing enteropathy [39].

### 4.4 Feasibility and outcomes of percutaneous closure

The technical feasibility of the use of Amplatzer devices and coils for closure of VVC in (adult) Fontan patients has been previously demonstrated in small cohort studies [[10], [11], [12],^19^,^20^,[27], [28], [29], [30], [31], [32]]. In our cohort, there was a beneficial (short-term) response to closure of the VVC in terms of oxygen saturation, comparable to previous reports [11,12,19,20,23,27,32]. This is in contrast to the findings of Poterucha et al., who did not only find no beneficial effect on oxygen saturation, but reported a decreased 5-year survival in patients who underwent closure of VVC in comparison to the patients who did not [10]. Our patients were followed during a mean maximal follow-up of 2 ± 1.5 (maximum of 5) years, in which no deaths occurred. In the cohort presented by Poterucha, the majority had an unfenestrated atrio-pulmonary Fontan and a large portion had heterotaxy syndrome, which has been associated with a poor long-term outcome after Fontan [10,40,41]. Our cohort represents a relatively good group of adult Fontan patients where the majority had a systemic left ventricle and no significant AV-valve regurgitation was present, all protective factors for long-term survival in the adult Fontan patients [42]. Additionally, Fontan pressures in our cohort were low in comparison to Poterucha et al. Of the papers reporting right sided pressures before and after closure, none reported a clinically relevant rise [11,23,32]. The other studies assessing short-term follow-up with beneficial results comparably report a high portion of systemic left ventricle patients and extracardiac conduits (ranging from 30 to 74 %), with low Fontan pressures prior to closure [11,12,19,20,23,27]. These differences might justify the differential results, emphasizing that patient selection on a case-to-case basis remains principal for the decision process to determine the optimal treatment strategy.

### 4.5 Recommendations for percutaneous closure

No direct recommendations regarding the indication criteria for percutaneous closure of VVC in Fontan patients can be made based on the results of this relatively small and ‘clinically stable’ patient cohort with low Fontan pressures. In literature, Masura et al. suggest using the same criteria for the closure of VVC in Fontan patients as are used for fenestration closure [23]. Bridges et al. suggest to refrain from fenestration closure in patients with systemic venous pressures >16 mmHg during test occlusion [43]. This is contrary to Pikhala et al., who recommended that it is safe to close the shunt in absence of a rise in systemic venous pressure (>4 mmHg) or reduction of venous oxygen saturation (>10 %) during test occlusion [44]. Masura et al. recommend performing a test occlusion and to close the fenestration in patients with systemic venous pressures <18 mm Hg, to use caution when pressures are ≥18 mm Hg and to refrain from closure when pressures are ≥20 mmHg [23]. Poterucha et al. recommend to carefully evaluate prior to considering closure of VVC in Fontan patients with atrio-pulmonary type Fontan, heterotaxy, and those with Fontan pressure above 18 mm Hg [10].

### 4.6 Gaps in literature

The literature review performed showed that although several studies on VVC in Fontan patients have been published, there still are important gaps in literature. The reported VVC prevalence is very heterogenous and therefore the true prevalence and its evolution with age and clinical status of the current Fontan population is still up for debate. Although some studies have eluded towards factors associated with VVC, there is limited data on the developmental and mechanistic factors and/or clinical risk stratification. Additionally, more data on adult-only cohorts is needed in this growing, and highly heterogenous and complex cohort. Data on resting oxygen saturation after closure and during long-term follow-up is scarce, whereas information on oxygen saturation during exercise is currently at large lacking. With the conflicting results on saturation, and limited information on follow-up, more data in the form of structured follow-up protocols and prospective international registries are needed to form an evidence-based recommendation for management strategy of VVC. The recommended routine follow-up and testing intervals in accordance with the American Heart Association guidelines are currently not widely implemented in Europe due to a range of factors [45]. The current study adds a small cohort to the scarcity of data currently available in adult Fontan patient. Moreover, we are the first to provide a systemic review focusing on the subject.

### 4.7 Study limitations

The results of this study should be interpreted in light of the small and heterogenous study population, and the retrospective, observational design. The study design inherently results in differential follow-up time. Only Fontan patients who underwent a closing procedure for VVC at adult age were included for analysis, possibly limiting the generalisation of results in a larger cohort. The indication for a percutaneous closing procedure was symptoms and/or significant exercise-related hypoxia in patients who were eligible to undergo catheterization, therefore selection could be biased towards a relatively good group of adult Fontan patients with relatively large collaterals. No exercise testing hemodynamic assessment was performed. Patient-reported symptoms were evaluated through subjective interviews, limiting the objectivity of this outcome measure in the observational nature of this study. The results may also have been influenced by healthcare providers' preferences in assessing the necessity and threshold for embolization (selection bias). Nevertheless, our report is one of few solely focusing on the growing group of young adult patients after Fontan palliation and underlines the high prevalence of VVC in this cohort, emphasizing the need for a longer-term follow up as well as mechanistic driven studies on this subject.

### 4.8 Future perspectives

Further research on developmental, clinical, genetic, and other (predictive) factors is mandatory to better understand the mechanism, clinical consequences, and optimal management strategies of VVC, especially in the growing and aging population of adult Fontan patients. The use of 4D flow Magnetic Resonance Imaging might also be useful to unravel a potential mismatch of Fontan tunnel size, turbulent flow profiles and the development of unloading VVC [[46], [47], [48]]. Optimally, closure strategy should be investigated in a large, prospective multicentre cohort including an invasive hemodynamic assessment during exercise. Patient selection remains key in the decision process to determine treatment strategy.

## 5 Conclusions

VVC in adult Fontan patients typically connect the tributaries of the vena cava inferior and/or superior with the pulmonary veins. A preserved ventricular function and low Fontan pressures do not exclude the presence of VVC. The percutaneous closure of VVC is technically feasible and safe and is associated with reduction of symptoms and a rise in the peripheral oxygen saturation at rest and peak exercise in this selected cohort. There are still several important gaps in literature, including the lack of data on long term follow-up in adult Fontan patients and the identification of driving factors for VVC development.

## Declaration of competing interest

The authors declare that they have no known competing financial interests or personal relationships that could have appeared to influence the work reported in this paper.
