# Supplementary material for: Percutaneous closure of veno-venous collaterals in adult patients with univentricular physiology after Fontan palliation: Single centre experience and systematic review
Source: Int J Cardiol Congenit Heart Dis. 2023 Oct 11;14:100479. doi: 10.1016/j.ijcchd.2023.100479 (PMC11657254; doi:10.1016/j.ijcchd.2023.100479)
Supplement: Multimedia component 1 [file mmc1.docx]

**Supplemental appendix**

**Supplement 1.** Search strategy

**PubMed**

(("venovenous collateral"[tw] OR "venovenous collaterals"[tw] OR "venovenous collateral*"[tw] OR "veno venous collateral"[tw] OR "veno venous collaterals"[tw] OR "veno venous collateral*"[tw] OR "pulmonary venous collateral"[tw] OR "pulmonary venous collaterals"[tw] OR "pulmonary venous collateral*"[tw] OR "Systemic venous collateral"[tw] OR "Systemic venous collaterals"[tw] OR "Systemic venous collateral*"[tw] OR "venovenous collateral"[title/abstract:~6] OR "venovenous collaterals"[title/abstract:~6] OR "veno venous collateral"[title/abstract:~6] OR "veno venous collaterals"[title/abstract:~6] OR "pulmonary venous collateral"[title/abstract:~6] OR "pulmonary venous collaterals"[title/abstract:~6] OR "Systemic venous collateral"[title/abstract:~6] OR "Systemic venous collaterals"[title/abstract:~6]) AND ("fontan circulation"[tw] OR "fontan circulations"[tw] OR "fontan circulatory"[tw] OR "fontan circulat*"[tw] OR "fontan circuit"[tw] OR "fontan circuits"[tw] OR "fontan circuit*"[tw] OR "Fontan Procedure"[Mesh] OR "Fontan Procedure"[tw] OR "Fontan Procedures"[tw] OR "Fontan Operation"[tw] OR "Fontan Operations"[tw] OR "Fontan Operat*"[tw] OR "Fontan palliation"[tw] OR "Fontan palliated"[tw] OR "cavopulmonary anastomosis"[tw] OR "cavopulmonary anastomoses"[tw] OR "cavopulmonary anastom*"[tw] OR "cavo pulmonary anastomosis"[tw] OR "cavo pulmonary anastomoses"[tw] OR "cavo pulmonary anastom*"[tw] OR "Bidirectional Glenn"[tw] OR "Bidirectional Glenn*"[tw] OR "Cavopulmonary Shunt"[tw] OR "Cavopulmonary Shunts"[tw] OR "Fontan Palliation"[tw] OR "Stage 2 Norwood Procedure"[tw] OR "Stage 3 Norwood Procedure"[tw] OR "Stage II Norwood Procedure"[tw] OR "Stage III Norwood Procedure"[tw] OR "Stage 2 Norwood Procedures"[tw] OR "Stage 3 Norwood Procedures"[tw] OR "Stage II Norwood Procedures"[tw] OR "Stage III Norwood Procedures"[tw] OR "fontan"[tw] OR "fontan*"[all fields] OR "Univentricular Heart"[Mesh] OR "Univentricular Heart"[tw] OR "Univentricular Hearts"[tw] OR "Univentricular Heart*"[tw] OR "Single Heart"[tw] OR "Single Hearts"[tw] OR "Single Heart*"[tw] OR "Single Ventricle"[tw] OR "Single Ventricles"[tw] OR "Single Ventricular"[tw] OR "Single Ventric*"[tw] OR "Hypoplastic left heart "[tw] OR "Hypoplastic right heart"[tw] OR "Hypoplastic left ventricle "[tw] OR "Hypoplastic right ventricle"[tw] OR "Hypoplastic left ventricles "[tw] OR "Hypoplastic right ventricles"[tw] OR "Hypoplastic left ventric*"[tw] OR "Hypoplastic right ventric*"[tw]))

Archivalia

OR (("Collateral Circulation"[Mesh] OR "Collateral Circulation"[Mesh] OR "collateral"[tw] OR "collaterals"[tw] OR "collateral*"[tw]) AND ("Pulmonary Veins"[mesh] OR "Pulmonary Veins"[tw] OR "Pulmonary Vein"[tw]))

OR "Heart Defects, Congenital/surgery"[mesh:noexp]

**Embase**

(("venovenous collateral".mp OR "venovenous collaterals".mp OR "venovenous collateral*".mp OR "veno venous collateral".mp OR "veno venous collaterals".mp OR "veno venous collateral*".mp OR "pulmonary venous collateral".mp OR "pulmonary venous collaterals".mp OR "pulmonary venous collateral*".mp OR "Systemic venous collateral".mp OR "Systemic venous collaterals".mp OR "Systemic venous collateral*".mp OR ("venovenous" ADJ5 "collateral").ti,ab OR ("venovenous" ADJ5 "collaterals").ti,ab OR ("veno" ADJ5 "venous" ADJ5 "collateral").ti,ab OR ("veno" ADJ5 "venous" ADJ5 "collaterals").ti,ab OR ("pulmonary" ADJ5 "venous" ADJ5 "collateral").ti,ab OR ("pulmonary" ADJ5 "venous" ADJ5 "collaterals").ti,ab OR ("Systemic" ADJ5 "venous" ADJ5 "collateral").ti,ab OR ("Systemic" ADJ5 "venous" ADJ5 "collaterals").ti,ab) AND ("fontan circulation".mp OR "fontan circulations".mp OR "fontan circulatory".mp OR "fontan circulat*".mp OR "fontan circuit".mp OR "fontan circuits".mp OR "fontan circuit*".mp OR "Fontan Procedure"/ OR "Fontan Procedure".mp OR "Fontan Procedures".mp OR "Fontan Operation".mp OR "Fontan Operations".mp OR "Fontan Operat*".mp OR "Fontan palliation".mp OR "Fontan palliated".mp OR "cavopulmonary connection"/ OR "cavopulmonary anastomosis".mp OR "cavopulmonary anastomoses".mp OR "cavopulmonary anastom*".mp OR "cavo pulmonary anastomosis".mp OR "cavo pulmonary anastomoses".mp OR "cavo pulmonary anastom*".mp OR "Bidirectional Glenn".mp OR "Bidirectional Glenn*".mp OR "Cavopulmonary Shunt".mp OR "Cavopulmonary Shunts".mp OR "Fontan Palliation".mp OR "Stage 2 Norwood Procedure".mp OR "Stage 3 Norwood Procedure".mp OR "Stage II Norwood Procedure".mp OR "Stage III Norwood Procedure".mp OR "Stage 2 Norwood Procedures".mp OR "Stage 3 Norwood Procedures".mp OR "Stage II Norwood Procedures".mp OR "Stage III Norwood Procedures".mp OR "fontan".mp OR "fontan*".mp OR "Heart Single Ventricle"/ OR "Univentricular Heart".mp OR "Univentricular Hearts".mp OR "Univentricular Heart*".mp OR "Single Heart".mp OR "Single Hearts".mp OR "Single Heart*".mp OR "Single Ventricle".mp OR "Single Ventricles".mp OR "Single Ventricular".mp OR "Single Ventric*".mp OR "Hypoplastic left heart ".mp OR "Hypoplastic right heart".mp OR "Hypoplastic left ventricle ".mp OR "Hypoplastic right ventricle".mp OR "Hypoplastic left ventricles ".mp OR "Hypoplastic right ventricles".mp OR "Hypoplastic left ventric*".mp OR "Hypoplastic right ventric*".mp))

- - NOT conference review.pt
  - NOT (conference review or conference abstract).pt
  - AND (conference abstract).pt

AND xxxx:2023.(sa_year)

**Web of Science**
TS=(("venovenous collateral" OR "venovenous collaterals" OR "venovenous collateral*" OR "veno venous collateral" OR "veno venous collaterals" OR "veno venous collateral*" OR "pulmonary venous collateral" OR "pulmonary venous collaterals" OR "pulmonary venous collateral*" OR "Systemic venous collateral" OR "Systemic venous collaterals" OR "Systemic venous collateral*" OR ("venovenous" NEAR/5 "collateral") OR ("venovenous" NEAR/5 "collaterals") OR ("veno" NEAR/5 "venous" NEAR/5 "collateral") OR ("veno" NEAR/5 "venous" NEAR/5 "collaterals") OR ("pulmonary" NEAR/5 "venous" NEAR/5 "collateral") OR ("pulmonary" NEAR/5 "venous" NEAR/5 "collaterals") OR ("Systemic" NEAR/5 "venous" NEAR/5 "collateral") OR ("Systemic" NEAR/5 "venous" NEAR/5 "collaterals")) AND ("fontan circulation" OR "fontan circulations" OR "fontan circulatory" OR "fontan circulat*" OR "fontan circuit" OR "fontan circuits" OR "fontan circuit*" OR "Fontan Procedure" OR "Fontan Procedure" OR "Fontan Procedures" OR "Fontan Operation" OR "Fontan Operations" OR "Fontan Operat*" OR "Fontan palliation" OR "Fontan palliated" OR "cavopulmonary connection" OR "cavopulmonary anastomosis" OR "cavopulmonary anastomoses" OR "cavopulmonary anastom*" OR "cavo pulmonary anastomosis" OR "cavo pulmonary anastomoses" OR "cavo pulmonary anastom*" OR "Bidirectional Glenn" OR "Bidirectional Glenn*" OR "Cavopulmonary Shunt" OR "Cavopulmonary Shunts" OR "Fontan Palliation" OR "Stage 2 Norwood Procedure" OR "Stage 3 Norwood Procedure" OR "Stage II Norwood Procedure" OR "Stage III Norwood Procedure" OR "Stage 2 Norwood Procedures" OR "Stage 3 Norwood Procedures" OR "Stage II Norwood Procedures" OR "Stage III Norwood Procedures" OR "fontan" OR "fontan*" OR "Heart Single Ventricle" OR "Univentricular Heart" OR "Univentricular Hearts" OR "Univentricular Heart*" OR "Single Heart" OR "Single Hearts" OR "Single Heart*" OR "Single Ventricle" OR "Single Ventricles" OR "Single Ventricular" OR "Single Ventric*" OR "Hypoplastic left heart " OR "Hypoplastic right heart" OR "Hypoplastic left ventricle " OR "Hypoplastic right ventricle" OR "Hypoplastic left ventricles " OR "Hypoplastic right ventricles" OR "Hypoplastic left ventric*" OR "Hypoplastic right ventric*"))

**Cochrane**

(("venovenous collateral" OR "venovenous collaterals" OR "venovenous collateral*" OR "veno venous collateral" OR "veno venous collaterals" OR "veno venous collateral*" OR "pulmonary venous collateral" OR "pulmonary venous collaterals" OR "pulmonary venous collateral*" OR "Systemic venous collateral" OR "Systemic venous collaterals" OR "Systemic venous collateral*" OR ("venovenous" NEAR/5 "collateral") OR ("venovenous" NEAR/5 "collaterals") OR ("veno" NEAR/5 "venous" NEAR/5 "collateral") OR ("veno" NEAR/5 "venous" NEAR/5 "collaterals") OR ("pulmonary" NEAR/5 "venous" NEAR/5 "collateral") OR ("pulmonary" NEAR/5 "venous" NEAR/5 "collaterals") OR ("Systemic" NEAR/5 "venous" NEAR/5 "collateral") OR ("Systemic" NEAR/5 "venous" NEAR/5 "collaterals")) AND ("fontan circulation" OR "fontan circulations" OR "fontan circulatory" OR "fontan circulat*" OR "fontan circuit" OR "fontan circuits" OR "fontan circuit*" OR "Fontan Procedure" OR "Fontan Procedure" OR "Fontan Procedures" OR "Fontan Operation" OR "Fontan Operations" OR "Fontan Operat*" OR "Fontan palliation" OR "Fontan palliated" OR "cavopulmonary connection" OR "cavopulmonary anastomosis" OR "cavopulmonary anastomoses" OR "cavopulmonary anastom*" OR "cavo pulmonary anastomosis" OR "cavo pulmonary anastomoses" OR "cavo pulmonary anastom*" OR "Bidirectional Glenn" OR "Bidirectional Glenn*" OR "Cavopulmonary Shunt" OR "Cavopulmonary Shunts" OR "Fontan Palliation" OR "Stage 2 Norwood Procedure" OR "Stage 3 Norwood Procedure" OR "Stage II Norwood Procedure" OR "Stage III Norwood Procedure" OR "Stage 2 Norwood Procedures" OR "Stage 3 Norwood Procedures" OR "Stage II Norwood Procedures" OR "Stage III Norwood Procedures" OR "fontan" OR "fontan*" OR "Heart Single Ventricle" OR "Univentricular Heart" OR "Univentricular Hearts" OR "Univentricular Heart*" OR "Single Heart" OR "Single Hearts" OR "Single Heart*" OR "Single Ventricle" OR "Single Ventricles" OR "Single Ventricular" OR "Single Ventric*" OR "Hypoplastic left heart " OR "Hypoplastic right heart" OR "Hypoplastic left ventricle " OR "Hypoplastic right ventricle" OR "Hypoplastic left ventricles " OR "Hypoplastic right ventricles" OR "Hypoplastic left ventric*" OR "Hypoplastic right ventric*")):ti,ab,kw

**Emcare**

(("venovenous collateral".mp OR "venovenous collaterals".mp OR "venovenous collateral*".mp OR "veno venous collateral".mp OR "veno venous collaterals".mp OR "veno venous collateral*".mp OR "pulmonary venous collateral".mp OR "pulmonary venous collaterals".mp OR "pulmonary venous collateral*".mp OR "Systemic venous collateral".mp OR "Systemic venous collaterals".mp OR "Systemic venous collateral*".mp OR ("venovenous" ADJ5 "collateral").ti,ab OR ("venovenous" ADJ5 "collaterals").ti,ab OR ("veno" ADJ5 "venous" ADJ5 "collateral").ti,ab OR ("veno" ADJ5 "venous" ADJ5 "collaterals").ti,ab OR ("pulmonary" ADJ5 "venous" ADJ5 "collateral").ti,ab OR ("pulmonary" ADJ5 "venous" ADJ5 "collaterals").ti,ab OR ("Systemic" ADJ5 "venous" ADJ5 "collateral").ti,ab OR ("Systemic" ADJ5 "venous" ADJ5 "collaterals").ti,ab) AND ("fontan circulation".mp OR "fontan circulations".mp OR "fontan circulatory".mp OR "fontan circulat*".mp OR "fontan circuit".mp OR "fontan circuits".mp OR "fontan circuit*".mp OR "Fontan Procedure"/ OR "Fontan Procedure".mp OR "Fontan Procedures".mp OR "Fontan Operation".mp OR "Fontan Operations".mp OR "Fontan Operat*".mp OR "Fontan palliation".mp OR "Fontan palliated".mp OR "cavopulmonary connection"/ OR "cavopulmonary anastomosis".mp OR "cavopulmonary anastomoses".mp OR "cavopulmonary anastom*".mp OR "cavo pulmonary anastomosis".mp OR "cavo pulmonary anastomoses".mp OR "cavo pulmonary anastom*".mp OR "Bidirectional Glenn".mp OR "Bidirectional Glenn*".mp OR "Cavopulmonary Shunt".mp OR "Cavopulmonary Shunts".mp OR "Fontan Palliation".mp OR "Stage 2 Norwood Procedure".mp OR "Stage 3 Norwood Procedure".mp OR "Stage II Norwood Procedure".mp OR "Stage III Norwood Procedure".mp OR "Stage 2 Norwood Procedures".mp OR "Stage 3 Norwood Procedures".mp OR "Stage II Norwood Procedures".mp OR "Stage III Norwood Procedures".mp OR "fontan".mp OR "fontan*".mp OR "Heart Single Ventricle"/ OR "Univentricular Heart".mp OR "Univentricular Hearts".mp OR "Univentricular Heart*".mp OR "Single Heart".mp OR "Single Hearts".mp OR "Single Heart*".mp OR "Single Ventricle".mp OR "Single Ventricles".mp OR "Single Ventricular".mp OR "Single Ventric*".mp OR "Hypoplastic left heart ".mp OR "Hypoplastic right heart".mp OR "Hypoplastic left ventricle ".mp OR "Hypoplastic right ventricle".mp OR "Hypoplastic left ventricles ".mp OR "Hypoplastic right ventricles".mp OR "Hypoplastic left ventric*".mp OR "Hypoplastic right ventric*".mp))

**Academic Search Premier**

((TI("venovenous collateral" OR "venovenous collaterals" OR "venovenous collateral*" OR "veno venous collateral" OR "veno venous collaterals" OR "veno venous collateral*" OR "pulmonary venous collateral" OR "pulmonary venous collaterals" OR "pulmonary venous collateral*" OR "Systemic venous collateral" OR "Systemic venous collaterals" OR "Systemic venous collateral*" OR ("venovenous" N5 "collateral") OR ("venovenous" N5 "collaterals") OR ("veno" N5 "venous" N5 "collateral") OR ("veno" N5 "venous" N5 "collaterals") OR ("pulmonary" N5 "venous" N5 "collateral") OR ("pulmonary" N5 "venous" N5 "collaterals") OR ("Systemic" N5 "venous" N5 "collateral") OR ("Systemic" N5 "venous" N5 "collaterals")) OR SU("venovenous collateral" OR "venovenous collaterals" OR "venovenous collateral*" OR "veno venous collateral" OR "veno venous collaterals" OR "veno venous collateral*" OR "pulmonary venous collateral" OR "pulmonary venous collaterals" OR "pulmonary venous collateral*" OR "Systemic venous collateral" OR "Systemic venous collaterals" OR "Systemic venous collateral*" OR ("venovenous" N5 "collateral") OR ("venovenous" N5 "collaterals") OR ("veno" N5 "venous" N5 "collateral") OR ("veno" N5 "venous" N5 "collaterals") OR ("pulmonary" N5 "venous" N5 "collateral") OR ("pulmonary" N5 "venous" N5 "collaterals") OR ("Systemic" N5 "venous" N5 "collateral") OR ("Systemic" N5 "venous" N5 "collaterals")) OR KW("venovenous collateral" OR "venovenous collaterals" OR "venovenous collateral*" OR "veno venous collateral" OR "veno venous collaterals" OR "veno venous collateral*" OR "pulmonary venous collateral" OR "pulmonary venous collaterals" OR "pulmonary venous collateral*" OR "Systemic venous collateral" OR "Systemic venous collaterals" OR "Systemic venous collateral*" OR ("venovenous" N5 "collateral") OR ("venovenous" N5 "collaterals") OR ("veno" N5 "venous" N5 "collateral") OR ("veno" N5 "venous" N5 "collaterals") OR ("pulmonary" N5 "venous" N5 "collateral") OR ("pulmonary" N5 "venous" N5 "collaterals") OR ("Systemic" N5 "venous" N5 "collateral") OR ("Systemic" N5 "venous" N5 "collaterals")) OR AB("venovenous collateral" OR "venovenous collaterals" OR "venovenous collateral*" OR "veno venous collateral" OR "veno venous collaterals" OR "veno venous collateral*" OR "pulmonary venous collateral" OR "pulmonary venous collaterals" OR "pulmonary venous collateral*" OR "Systemic venous collateral" OR "Systemic venous collaterals" OR "Systemic venous collateral*" OR ("venovenous" N5 "collateral") OR ("venovenous" N5 "collaterals") OR ("veno" N5 "venous" N5 "collateral") OR ("veno" N5 "venous" N5 "collaterals") OR ("pulmonary" N5 "venous" N5 "collateral") OR ("pulmonary" N5 "venous" N5 "collaterals") OR ("Systemic" N5 "venous" N5 "collateral") OR ("Systemic" N5 "venous" N5 "collaterals"))) AND (TI("fontan circulation" OR "fontan circulations" OR "fontan circulatory" OR "fontan circulat*" OR "fontan circuit" OR "fontan circuits" OR "fontan circuit*" OR "Fontan Procedure" OR "Fontan Procedure" OR "Fontan Procedures" OR "Fontan Operation" OR "Fontan Operations" OR "Fontan Operat*" OR "Fontan palliation" OR "Fontan palliated" OR "cavopulmonary connection" OR "cavopulmonary anastomosis" OR "cavopulmonary anastomoses" OR "cavopulmonary anastom*" OR "cavo pulmonary anastomosis" OR "cavo pulmonary anastomoses" OR "cavo pulmonary anastom*" OR "Bidirectional Glenn" OR "Bidirectional Glenn*" OR "Cavopulmonary Shunt" OR "Cavopulmonary Shunts" OR "Fontan Palliation" OR "Stage 2 Norwood Procedure" OR "Stage 3 Norwood Procedure" OR "Stage II Norwood Procedure" OR "Stage III Norwood Procedure" OR "Stage 2 Norwood Procedures" OR "Stage 3 Norwood Procedures" OR "Stage II Norwood Procedures" OR "Stage III Norwood Procedures" OR "fontan" OR "fontan*" OR "Heart Single Ventricle" OR "Univentricular Heart" OR "Univentricular Hearts" OR "Univentricular Heart*" OR "Single Heart" OR "Single Hearts" OR "Single Heart*" OR "Single Ventricle" OR "Single Ventricles" OR "Single Ventricular" OR "Single Ventric*" OR "Hypoplastic left heart " OR "Hypoplastic right heart" OR "Hypoplastic left ventricle " OR "Hypoplastic right ventricle" OR "Hypoplastic left ventricles " OR "Hypoplastic right ventricles" OR "Hypoplastic left ventric*" OR "Hypoplastic right ventric*") OR SU("fontan circulation" OR "fontan circulations" OR "fontan circulatory" OR "fontan circulat*" OR "fontan circuit" OR "fontan circuits" OR "fontan circuit*" OR "Fontan Procedure" OR "Fontan Procedure" OR "Fontan Procedures" OR "Fontan Operation" OR "Fontan Operations" OR "Fontan Operat*" OR "Fontan palliation" OR "Fontan palliated" OR "cavopulmonary connection" OR "cavopulmonary anastomosis" OR "cavopulmonary anastomoses" OR "cavopulmonary anastom*" OR "cavo pulmonary anastomosis" OR "cavo pulmonary anastomoses" OR "cavo pulmonary anastom*" OR "Bidirectional Glenn" OR "Bidirectional Glenn*" OR "Cavopulmonary Shunt" OR "Cavopulmonary Shunts" OR "Fontan Palliation" OR "Stage 2 Norwood Procedure" OR "Stage 3 Norwood Procedure" OR "Stage II Norwood Procedure" OR "Stage III Norwood Procedure" OR "Stage 2 Norwood Procedures" OR "Stage 3 Norwood Procedures" OR "Stage II Norwood Procedures" OR "Stage III Norwood Procedures" OR "fontan" OR "fontan*" OR "Heart Single Ventricle" OR "Univentricular Heart" OR "Univentricular Hearts" OR "Univentricular Heart*" OR "Single Heart" OR "Single Hearts" OR "Single Heart*" OR "Single Ventricle" OR "Single Ventricles" OR "Single Ventricular" OR "Single Ventric*" OR "Hypoplastic left heart " OR "Hypoplastic right heart" OR "Hypoplastic left ventricle " OR "Hypoplastic right ventricle" OR "Hypoplastic left ventricles " OR "Hypoplastic right ventricles" OR "Hypoplastic left ventric*" OR "Hypoplastic right ventric*") OR KW("fontan circulation" OR "fontan circulations" OR "fontan circulatory" OR "fontan circulat*" OR "fontan circuit" OR "fontan circuits" OR "fontan circuit*" OR "Fontan Procedure" OR "Fontan Procedure" OR "Fontan Procedures" OR "Fontan Operation" OR "Fontan Operations" OR "Fontan Operat*" OR "Fontan palliation" OR "Fontan palliated" OR "cavopulmonary connection" OR "cavopulmonary anastomosis" OR "cavopulmonary anastomoses" OR "cavopulmonary anastom*" OR "cavo pulmonary anastomosis" OR "cavo pulmonary anastomoses" OR "cavo pulmonary anastom*" OR "Bidirectional Glenn" OR "Bidirectional Glenn*" OR "Cavopulmonary Shunt" OR "Cavopulmonary Shunts" OR "Fontan Palliation" OR "Stage 2 Norwood Procedure" OR "Stage 3 Norwood Procedure" OR "Stage II Norwood Procedure" OR "Stage III Norwood Procedure" OR "Stage 2 Norwood Procedures" OR "Stage 3 Norwood Procedures" OR "Stage II Norwood Procedures" OR "Stage III Norwood Procedures" OR "fontan" OR "fontan*" OR "Heart Single Ventricle" OR "Univentricular Heart" OR "Univentricular Hearts" OR "Univentricular Heart*" OR "Single Heart" OR "Single Hearts" OR "Single Heart*" OR "Single Ventricle" OR "Single Ventricles" OR "Single Ventricular" OR "Single Ventric*" OR "Hypoplastic left heart " OR "Hypoplastic right heart" OR "Hypoplastic left ventricle " OR "Hypoplastic right ventricle" OR "Hypoplastic left ventricles " OR "Hypoplastic right ventricles" OR "Hypoplastic left ventric*" OR "Hypoplastic right ventric*") OR AB("fontan circulation" OR "fontan circulations" OR "fontan circulatory" OR "fontan circulat*" OR "fontan circuit" OR "fontan circuits" OR "fontan circuit*" OR "Fontan Procedure" OR "Fontan Procedure" OR "Fontan Procedures" OR "Fontan Operation" OR "Fontan Operations" OR "Fontan Operat*" OR "Fontan palliation" OR "Fontan palliated" OR "cavopulmonary connection" OR "cavopulmonary anastomosis" OR "cavopulmonary anastomoses" OR "cavopulmonary anastom*" OR "cavo pulmonary anastomosis" OR "cavo pulmonary anastomoses" OR "cavo pulmonary anastom*" OR "Bidirectional Glenn" OR "Bidirectional Glenn*" OR "Cavopulmonary Shunt" OR "Cavopulmonary Shunts" OR "Fontan Palliation" OR "Stage 2 Norwood Procedure" OR "Stage 3 Norwood Procedure" OR "Stage II Norwood Procedure" OR "Stage III Norwood Procedure" OR "Stage 2 Norwood Procedures" OR "Stage 3 Norwood Procedures" OR "Stage II Norwood Procedures" OR "Stage III Norwood Procedures" OR "fontan" OR "fontan*" OR "Heart Single Ventricle" OR "Univentricular Heart" OR "Univentricular Hearts" OR "Univentricular Heart*" OR "Single Heart" OR "Single Hearts" OR "Single Heart*" OR "Single Ventricle" OR "Single Ventricles" OR "Single Ventricular" OR "Single Ventric*" OR "Hypoplastic left heart " OR "Hypoplastic right heart" OR "Hypoplastic left ventricle " OR "Hypoplastic right ventricle" OR "Hypoplastic left ventricles " OR "Hypoplastic right ventricles" OR "Hypoplastic left ventric*" OR "Hypoplastic right ventric*")))
